# Effect of Changes in Plastic Flow During Non-Steady State Deformation on Force Behavior in Micro-Extrusion of Pure Copper †

**DOI:** 10.3390/ma19071374

**Published:** 2026-03-30

**Authors:** Keisuke Sugiyama, Masato Ito, Kenichi Yaguchi, Tatsuya Funazuka, Tomomi Shiratori

**Affiliations:** 1 Mitsubishi Materials Corporation, 7-147 Shimoishito, Kitamoto 364-0028, Saitama, Japan; itol@mmc.co.jp (M.I.); yaguchi@mmc.co.jp (K.Y.); 2 Department of Mechanical Engineering, Toyama University, 3190 Gofuku, Toyama-shi 930-8555, Toyama, Japan; funazuka@eng.u-toyama.ac.jp (T.F.); shira@eng.u-toyama.ac.jp (T.S.)

**Keywords:** micro-forming, deformation behavior, size effect, plastic deformation, pure copper

## Abstract

In recent years, copper-based conductive and heat dissipation components have required fine structures for miniaturization and enhanced functionality. Micro-forming is an excellent processing method characterized by high productivity and suitability for mass production. Since small workpieces can be formed within a short stroke in micro-extrusion, it is important to understand the deformation behavior immediately after the start of extrusion. However, before steady state is attained, the evolution of microstructure and plastic flow with stroke progression during non-steady-state deformation has not yet been sufficiently clarified. In this study, to investigate the effect of changes in plastic flow on force behavior, micro-extrusion tests were conducted using pure copper. The geometric and crystallographic characteristics of the deformation structure were then analyzed. The extrusion force behavior exhibited three distinct stages, including a peak of the force. The force peak was attributed to changes in plastic flow associated with the deformation structure formed at the sample tip immediately after the start of extrusion. This change leads to the evolution of the effective extrusion ratio, which significantly influences the force response during non-steady-state deformation.

## 1. Introduction

Copper is widely used for heat-dissipation components and connector terminals because of its excellent thermal and electrical conductivities. In recent years, the electrification of automobiles and the growing demand for data centers driven by the widespread adoption of AI have accelerated [1,2,3]. Accordingly, demand for these components is expected to increase further in the coming years. These components are increasingly required to incorporate fine, complex geometries to enable miniaturization and enhanced functionality. Therefore, it is necessary to establish copper forming processes that can accommodate micro- and meso-scale fabrication while ensuring high productivity suitable for mass production. Various metal processing routes are available, including machining, additive manufacturing, and plastic forming. Plastic forming is one of the most widely used methods. Plastic forming is a manufacturing process in which a metal workpiece is shaped using dies. Once the die is prepared, identical parts can be produced in large quantities within short cycle times, making the process well-suited to high-volume production. Because the process entails low material loss, it enables a high level of productivity. In addition, the imposed plastic strain may induce work hardening, potentially improving the mechanical performance of the formed parts [4]. Accordingly, there is a growing need to establish micro-forming technology that extends plastic forming processes to the micro- and meso-scales for copper materials.

In micro-forming, challenges arise from size effects [5,6,7,8]. Here, “size effects” refer to the phenomenon that, as the dimensions of the workpiece decrease, the influences of microstructural factors (e.g., grain size and crystal orientation) and tribological conditions (e.g., die-surface roughness and coatings) on formability (e.g., forming force and dimensional accuracy) become relatively more pronounced. In micro-forming, it is necessary to accurately understand such size effects and to appropriately determine the processing conditions accordingly [9,10,11,12,13,14]. To address challenges attributable to size effects, numerous studies have been reported on micro-extrusion and related micro-forming processes. For example, Chan et al. conducted compression tests on pure copper and found that the effects of anisotropy of each grain on deformation behaviors became more pronounced as the ratio of sample size to grain size decreased [15]. They also reported that the simulation results of the finite element method model considering anisotropy were in good agreement with the experimental results in terms of flow stress behavior and non-uniform deformation. Krishnan et al. examined the interplay between frictional conditions and size effects in micro-extrusion, showing that hard die-surface coatings can help stabilize interfacial friction during forming; in particular, diamond-like carbon deposited silicon (DLC–Si) coatings were effective in reducing the maximum forming force [16]. In addition, Funazuka et al. reported through forward–backward micro-extrusion tests using aluminum alloys that the surface characteristics of the die changed the force behavior and had a significant impact on product shape [9]. Many of the published studies related to micro-extrusion focus on how variations in process parameters affect the product shape and the maximum forming force, as well as deformation behavior after the process has reached a steady state. In contrast, before steady state is attained, the evolution of microstructure and plastic flow with stroke progression during non-steady-state deformation has not yet been sufficiently clarified. It also remains unclear how these changes influence the force response. In micro-extrusion, small workpieces can potentially be formed within a short stroke. Therefore, in the manufacturing of products by micro-extrusion, a significant portion of the stroke required for product formation may be dominated by non-steady-state deformation before steady-state deformation is established. Furthermore, the maximum extrusion force during forming is often recorded during this non-steady-state stage. An accurate understanding of deformation behavior, including non-steady-state deformation, is crucial for process design in micro-extrusion. Based on this understanding, appropriate processing conditions must be established to address key challenges such as reducing the maximum extrusion force and improving die life.

We have investigated the non-steady-state deformation stage of cold micro-extrusion of pure copper, and the formation of characteristic deformation structures was observed [14]. However, quantitative evaluation remains insufficient, and the mechanisms underlying the formation of these characteristic deformation structures, as well as their evolution during stroke progression, have not yet been fully elucidated. In this paper, the changes in these deformation structures formed during non-steady-state deformation were systematically analyzed using quantitative evaluation methods, and the mechanisms of their formation and evolution were examined. The geometric and crystallographic characteristics of the deformed microstructure formed at strokes of 2.00 mm or less were analyzed. In addition, samples formed at multiple intermediate strokes were analyzed to elucidate how progressive changes in microstructure correlated with the observed force behavior.

## 2. Experimental Procedure

**Figure 1:**
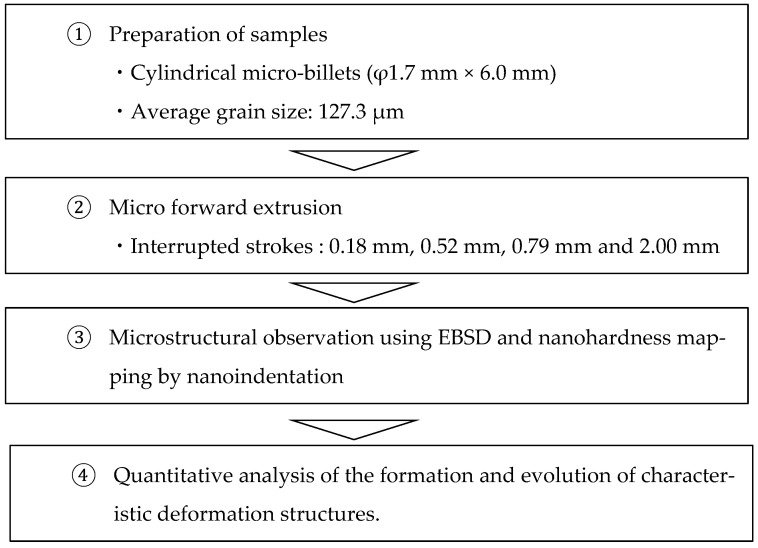
The research workflow.

**Figure 2:**
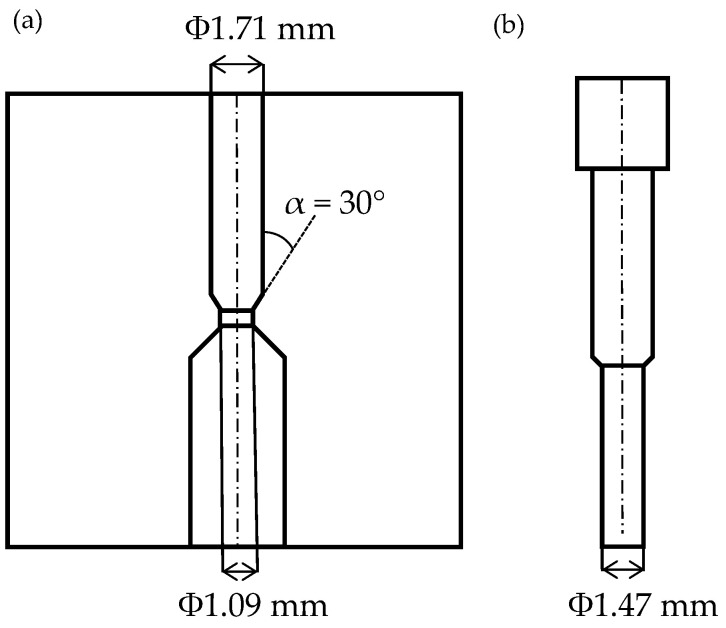
Key dimensions of the (**a**) die and (**b**) punch [14].

Figure 1 shows the research workflow. The extrusion test conditions were set to be equivalent to those used in the authors’ previous studies [14]. The test material was pure copper, which was cold-rolled to a reduction ratio of 40% and subsequently heat-treated at 550 °C for 3 h, followed by water quenching, to obtain an average grain size of 127.3 µm. The material was machined to cut out a cylindrical sample with a diameter of 1.7 mm and a height of 6.0 mm. For the micro-extrusion process, forward and backward extrusion was used. Figure 2 shows a schematic diagram of the die and punch. The die has a split structure to remove the sample after extrusion and consists of three sections: a container section, a tapered section, and a bearing section. The inside diameter of the container is 1.71 mm, the inside diameter of the bearing is 1.09 mm, and the angle (die semi-angle) α between the die face of the tapered section and the extrusion axis direction is 30°. The die was coated with diamond-like carbon (DLC), which has been reported to provide a lubricating effect even in the micro-extrusion of copper materials [13,16]. The punch tip diameter was 1.47 mm, and the cross-sectional area reduction rate was set to 59.4% for forward extrusion and 73.9% for backward extrusion. A mirror-finished punch with a ground surface and no DLC coating was used. Micro-extrusion was performed at room temperature under the following conditions: stroke velocity 0.10 mm/s, maximum stroke 2.00 mm, hold time 1.0 s, and lubricant kinematic viscosity 429 mm^2^/s. To observe microstructure changes with the progression of stroke, interrupted tests were conducted by stopping the punch at predefined stroke positions less than 2.00 mm.

**Figure 3:**
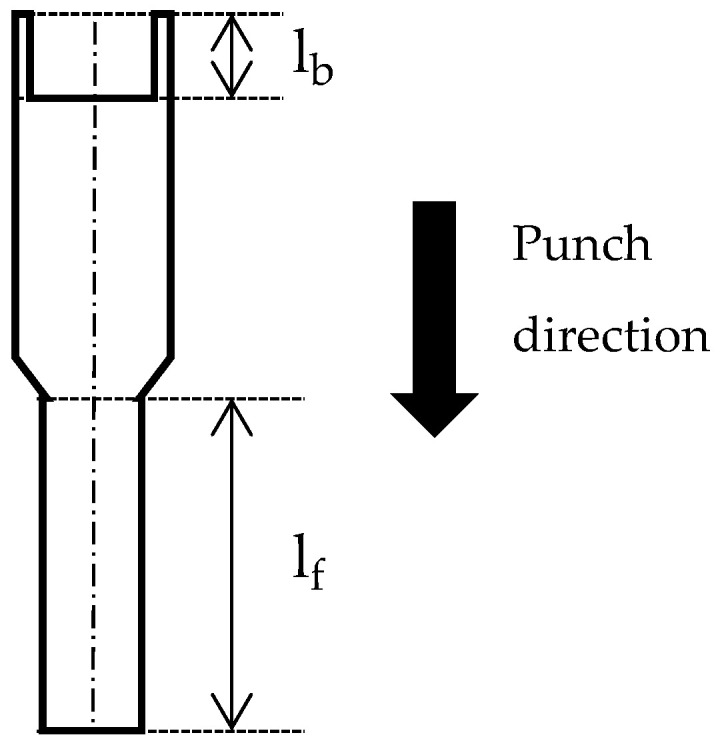
Schematic drawing of the extruded sample shape and evaluated lengths.

After the micro-extrusion tests, the samples were sectioned parallel to the extrusion direction. The cross-sections were examined by optical microscopy to evaluate the formed geometry and by electron backscatter diffraction (EBSD) to characterize the crystal orientation. Figure 3 shows a schematic drawing of the extruded sample shape and evaluates the forward extrusion length (lf) and backward extrusion length (lb). From the EBSD data, an image quality (IQ) map, an inverse pole figure (IPF) map, a kernel average misorientation (KAM) map, and a unique grain color (UGC) map were obtained. The UGC map is a map in which each grain is assigned a random color. In this study, boundaries with a misorientation angle of 5° or more were defined as grain boundaries in the UGC map. Furthermore, nanoindentation tests were performed on the cross-sections of the samples. The tests were conducted on a regular grid with a fixed spacing to obtain a nanohardness map. A Berkovich indenter was used, and the tests were conducted under force control up to a maximum force of 5.0 mN, with a hold time of 1.0 s at the maximum force.

## 3. Result

**Figure 4:**
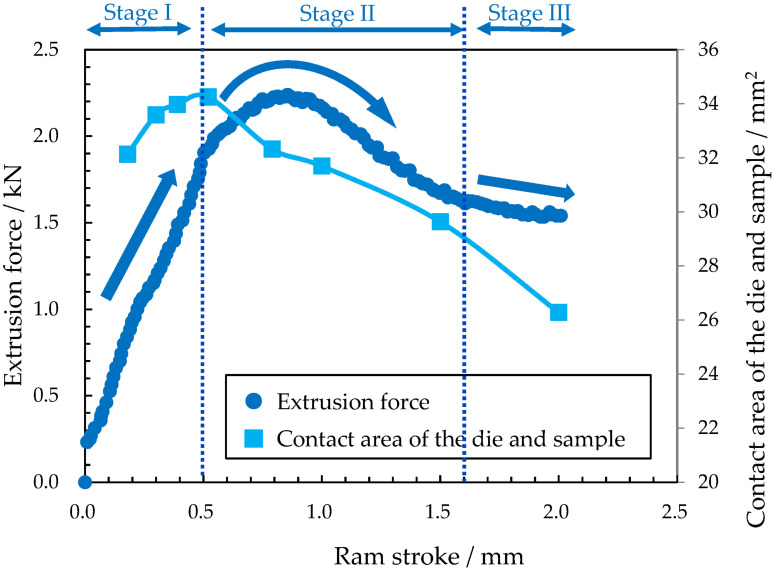
Extrusion force–stroke curves and die–sample contact area dependent on sample shape.

Figure 4 shows the extrusion force–stroke curves and die–sample contact area dependent on sample shape. The contact area was estimated from the directly measured sample dimensions obtained from interrupted tests. The maximum force (2.24 kN) occurred at a stroke of 0.86 mm, whereas the contact area reached its maximum (34.2 mm^2^) at a stroke of 0.52 mm. The latter stroke corresponded to the moment when the sample tip reached the outlet of the bearing section. Focusing on the force–stroke curve, the force behavior can be divided into the following three stages:

- Stage I—a linear increase;
- Stage II—a peak;
- Stage III—a gradual decrease.

In general, in forward extrusion, an increase in the deformation volume near the sample tip causes the force to increase, whereas the subsequent decrease in the contact area between the sample and the container section of the die leads to a reduction in force [17]. The force behaviors in Stages I and III were considered to correspond to these phenomena, respectively. In contrast, the force peak observed in Stage II cannot be explained by such a simple mechanism. This is evident because the strokes corresponding to the maximum contact area and the maximum force did not coincide, and the force continued to increase even after the contact area began to decrease. It is worth noting that, in Stage III, the slope of the force–stroke curve became approximately constant, indicating that the deformation approached a steady state. These results suggest that the force peak observed in Stage II could be attributed to stroke-dependent changes in microstructure and plastic flow during non-steady-state deformation.

**Figure 5:**
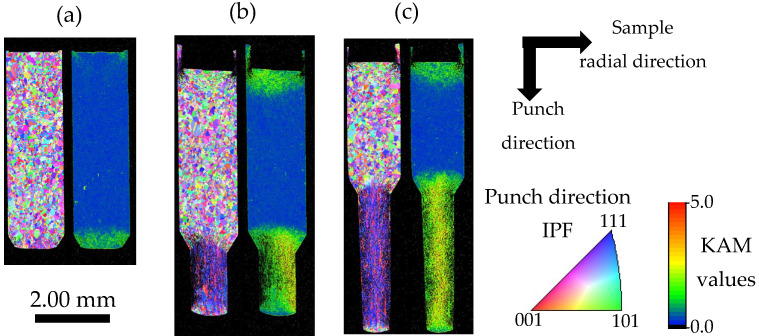
IPF map of punch direction and KAM map at strokes of (**a**) 0.18 mm, (**b**) 0.79 mm, (**c**) 2.00 mm.

**Figure 6:**
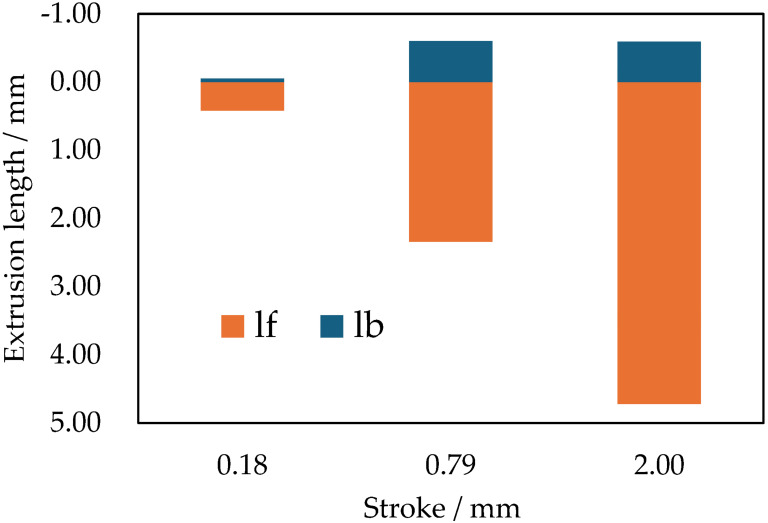
Extrusion length at strokes of 0.18 mm, 0.79 mm and 2.00 mm.

Figure 5 shows IPF maps and KAM maps of the cross-sections of samples extruded to strokes of (a) 0.18 mm, (b) 0.79 mm, and (c) 2.00 mm. Figure 6 shows l_b_ and l_f_ at each stroke. Although the reduction rate in the backward direction is 14.5% higher than that in the forward direction, all samples at every stroke exhibit both forward- and backward-extruded regions. High KAM values were observed near both regions, indicating the introduction of plastic strain. In the forward region, a fiber texture with preferential orientations close to ⟨001⟩ and ⟨111⟩ was observed in the IPF maps. In contrast, in most of the region between the forward- and backward-extruded regions, the initial microstructure is largely retained, with low KAM values. This indicates that plastic deformation in this region is much less than that in the forward- and backward-extruded regions, with no large-scale plastic flow along the sample length. Focusing on the stroke-dependent evolution of extrusion length (Figure 6), both l_f_ and l_b_ increase from a stroke of 0.18 mm to 0.79 mm. However, from 0.79 mm to 2.00 mm, l_f_ increases predominantly, whereas l_b_ exhibits little change. While only l_f_ increases, the extrusion force decreases. These results suggest that, during extrusion, changes in plastic flow and microstructure in the forward region reduce the force required to extrude the sample forward. To fully understand this phenomenon, detailed observations focusing on the forward-extruded region were conducted, as described in the next section.

**Figure 7:**
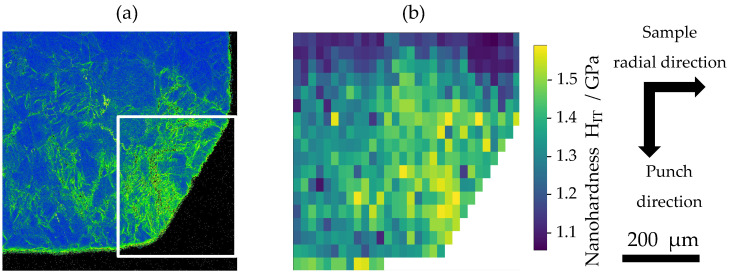
(**a**) KAM map and (**b**) hardness map at a stroke of 0.18 mm [14]. The KAM map shows a concentration of deformation. The nanohardness map shows a heterogeneous distribution of work hardening.

Figure 7 shows (a) the KAM map and (b) the nanohardness map of the tip of the cross-sections of samples extruded to strokes of 0.18 mm [14]. At a stroke of 0.18 mm, i.e., immediately after the start of the test, a heterogeneous distribution of KAM values is observed. As also shown in Figure 5, the KAM values are low within the container section, whereas they are relatively high in the region that has entered the tapered section. Furthermore, the KAM distribution remains heterogeneous even within the tapered section. As indicated by the white frames in the figure, high KAM values are concentrated near the die–sample contact interface, suggesting that plastic deformation is localized in this region. In the nanohardness map, an increase in nanohardness due to work hardening is observed. The nanohardness distribution is also heterogeneous and corresponds well to the KAM map. These results indicate that, immediately after the start of the test, deformation is concentrated near the die–sample contact interface of the sample tip, resulting in a hardness gradient in the radial direction within the tapered section.

**Figure 8:**
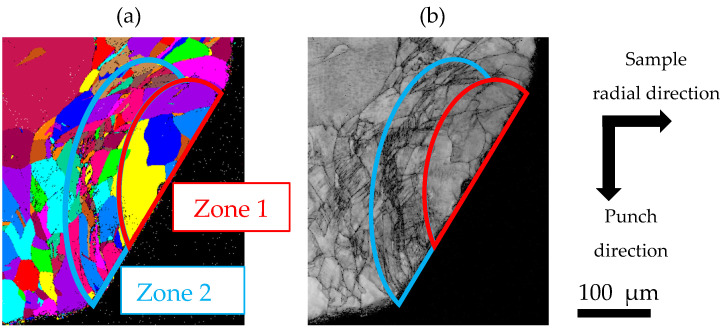
(**a**) UGC map and (**b**) IQ map at a stroke of 0.18 mm, corresponding to the area indicated by a white square in Figure 7a. Dark regions in the IQ map are associated with reduced crystallinity caused by severe plastic deformation.

Figure 8 shows (a) a UGC map and (b) an IQ map at a stroke of 0.18 mm, corresponding to the area indicated by the white frame in Figure 7a. The authors noted that a characteristic deformation structure forms immediately after the start of the test and, from qualitative assessment, suggested that this structure may influence the force response [14]. In this paper, we analyze this deformation structure in detail to quantitatively evaluate its formation mechanism, its evolution, and its effects on the force response. During plastic deformation, grains can change their morphology or become refined. Careful observation of grain morphology and size may be effective for evaluating the state of plastic flow occurring within the sample. In the UGC map, two zones with distinct characteristics are observed. Zone 1 is closest to the die–sample contact interface and has a dome-like shape. In this zone, the grains are refined relative to the initial grain size, although no pronounced morphological features are evident. In contrast, Zone 2, which surrounds Zone 1, contains many grains that are much finer or more flattened than those in Zone 1. In the IQ map, a streak-like band with markedly low IQ values is observed in Zone 2. The IQ parameter reflects the quality of the EBSD pattern and can be used as a qualitative indicator of local lattice distortion [18]. Accordingly, the low-IQ band in Zone 2 indicates pronounced lattice distortion induced by severe plastic flow. These results imply the occurrence of curved and severe localized plastic flow in Zone 2 at a stroke of 0.18 mm. The plastic flow in Zone 2 is curved, with the curvature characterized by the dome-shaped morphology of Zone 1. This behavior indicates that Zone 1 may act as a dead-metal-like zone, effectively constraining and directing the path of the surrounding plastic flow. We refer to Zone 1 and Zone 2, which exhibit distinct characteristics, as the dead-metal-like zone (DMLZ) and the localized-deformation zone (LDZ), respectively.

## 4. Discussion

In the previous section, it was revealed that heterogeneous deformation develops in the tapered section immediately after the start of the extrusion test, and that two distinct deformation microstructures (DMLZ and LDZ) form near the die–sample contact interface. In this section, the formation mechanisms of these microstructures and their evolution with stroke progression are analyzed to discuss the effect of changes in plastic flow on the force behavior.

### 4.1. Formation Mechanisms of the Deformation Microstructures

**Figure 9:**
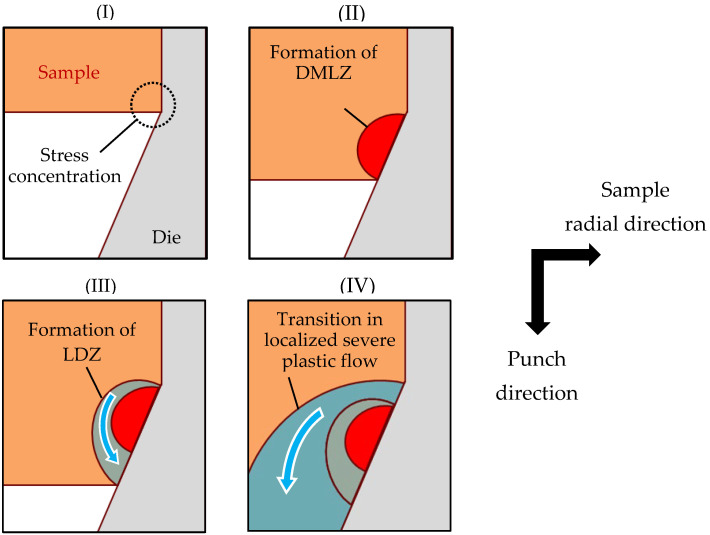
Schematic illustration of the formation mechanisms of the two deformation microstructures (DMLZ and LDZ) and their evolution with stroke progression.

Figure 9 shows a schematic illustration of the formation mechanisms of the two deformation microstructures and their evolution with stroke progression. Immediately after the start of the test, stress concentrates near the outer periphery of the forward end of the sample (I). As a result, heterogeneous deformation develops at the sample tip, and a work-hardened deformation microstructure forms near the die–sample contact interface (II). This microstructure is considered to correspond to DMLZ. During the formation of this microstructure, fresh surfaces are exposed, and local adhesion is expected to occur when these fresh surfaces come into contact with the die. Owing to its locally increased hardness and localized adhesion to the die, DMLZ can act as a resistance to subsequent plastic flow in the surrounding material. In contrast, the adjacent region is expected to deform more readily because it experiences a relatively smaller degree of work hardening and is less affected by adhesion. After the formation of DMLZ, such regions may deform preferentially, leading to a severely localized plastic flow along DMLZ. The LDZ is considered to be formed through this process (III). Because the plastic flow detours around Zone 1, the deformation is more severe than that expected for the ideal flow along the die taper. Consequently, due to the formation of DMLZ, the effective extrusion ratio becomes higher than the nominal extrusion ratio defined by the die geometry. Furthermore, because the LDZ can act as an additional resistance to subsequent plastic flow, the severely localized plastic flow is expected to transition radially inward as the stroke progresses (IV). As a result, the effective extrusion ratio is expected to continue increasing.

### 4.2. Relationship Between Microstructural Evolution and Force Behavior

**Figure 10:**
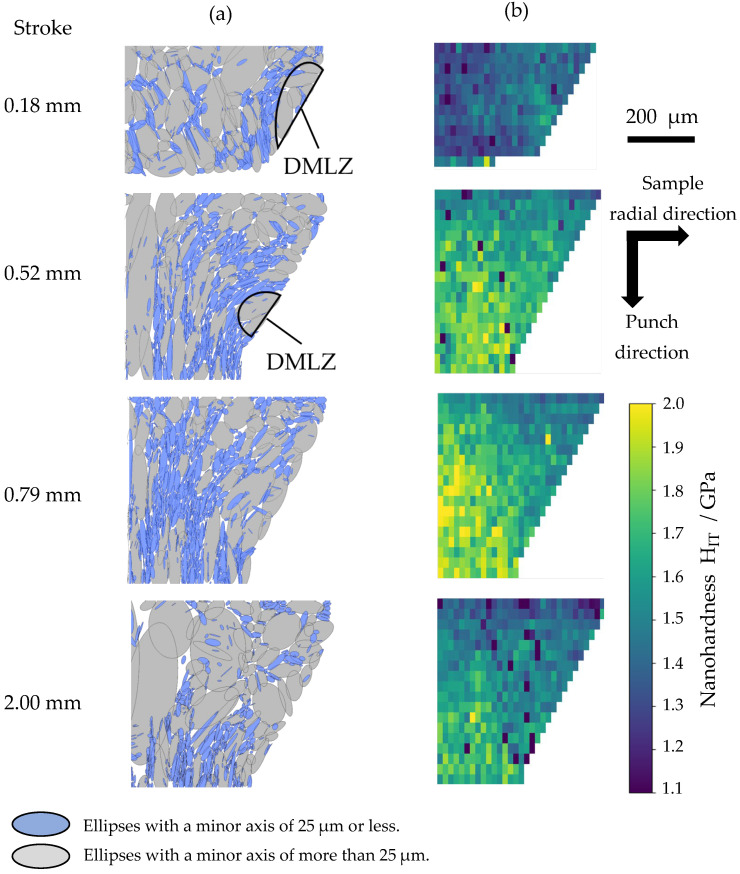
(**a**) Ellipse-approximated grain maps, and (**b**) Nanohardness maps in the tapered section at strokes of 0.18, 0.52, 0.79, and 2.00 mm.

Figure 10 shows (a) ellipse-approximated grain maps and (b) nanohardness maps in the tapered section at strokes of 0.18, 0.52, 0.79, and 2.00 mm. The ellipse-approximated grain map was obtained by importing the UGC map into ImageJ (v1.54p) and, using the MorphoLibJ plugin (v1.6.5), binarizing and labeling each grain, followed by calculating an inertia ellipse for each grain based on the second central moments. In evaluating microstructural evolution with stroke progression, direct comparison of the UGC maps provides only a qualitative assessment. In contrast, converting the UGC maps into ellipse-approximated grain maps enables quantitative evaluation of grain refinement and grain flattening associated with plastic deformation. The blue highlights in the map indicate ellipses with a minor-axis length of 25 µm or less. These highlighted regions correspond to areas where sufficient grain refinement or grain flattening has occurred relative to the initial grain.

First, we focus on the microstructural evolution up to the maximum force, where blue-highlighted grains are distributed around the DMLZ at a stroke of 0.18 mm. This distribution corresponds to the LDZ defined in Figure 8. Immediately after the force enters Stage II (0.52 mm), this distribution expands radially inward. Around the maximum force (0.79 mm), it extends further toward the sample axis. These microstructural changes suggest a transition of severely localized plastic flow over this stroke range. DMLZ does not remain stationary like a typical dead-metal zone; instead, it migrates forward as the stroke progresses and is extruded from the taper section around the maximum force. The DMLZ forms immediately after the start of the test and is observed in the taper section over a limited stroke. Nevertheless, plastic flow after its extrusion from the taper section is also influenced by the forward-located DMLZ. The nanohardness distribution exhibits a corresponding expansion of a significant work-hardened region from near the die–sample contact interface toward the inner radial direction. This evolution of the nanohardness distribution is considered to result from severe deformation imposed by the increase in the effective extrusion ratio associated with the transition in plastic flow. As can be seen from the nanohardness distribution at a stroke of 0.79 mm, around the maximum force, the region near the exit of the tapered section is largely occupied by a markedly expanded work-hardened region. The formation of such a structure suggests that material flow near the taper exit is impeded, leading to local stagnation. The extrusion force increases until sufficient stress is reached to push this hardened structure forward, which is considered to correspond to the peak in the extrusion force (maximum extrusion force). The force increase that does not coincide with the evolution of the die–sample contact area can therefore be attributed to this phenomenon. It should be noted that a quantitative evaluation of the effective extrusion ratio has not been established in the present study, and its variation with stroke progression has not yet been fully captured. Establishing a quantitative model for the effective extrusion ratio, based on the geometric characteristics of the DMLZ and LDZ, remains an important subject for future work. Next, focus on the microstructural evolution after the maximum force. From 0.79 to 2.00 mm, the fraction of refined or flattened grains in the tapered section decreases, while relatively coarse grains flow into the tapered section. Simultaneously, the nanohardness in the tapered section decreases overall. These results suggest that, after the significant work-hardened microstructure has largely filled the tapered section, the deformation mode shifts to a mode in which the microstructure is pushed forward. This shift reduces the effective extrusion ratio, thereby leading to a rapid decrease in force. In summary, the evolving state of plastic flow during non-steady-state deformation is closely linked to the force behavior, and the force peak originates from changes in the effective extrusion ratio associated with this evolving state of plastic flow. These findings suggest that, to reduce the maximum extrusion force and improve die life, it is essential to suppress the increase in the effective extrusion ratio and thereby mitigate the peak of force during processing. In particular, controlling the formation of the DMLZ at the early stage of extrusion is critical. This may be achieved through optimization of processing conditions, such as the application of appropriate coatings to the die and/or workpiece, as well as the introduction of pre-processing steps, provided that the associated increase in process complexity remains acceptable.

## 5. Conclusions

To investigate the effect of changes in plastic flow on force behavior, micro-extrusion tests were conducted using pure copper. As a result of analyzing the geometric and crystallographic characteristics of the deformation structure, the following findings were obtained.

1. The extrusion force behavior consisted of three stages: a linear increase in force (Stage I), a peak of force (Stage II), and a gradual decrease in force (Stage III). The maximum force was recorded at the peak of Stage II, but the stroke at this point differed from the stroke at which the contact area between the sample and the die reached its maximum.
2. Immediately after the start of the extrusion test, heterogeneous deformation occurred in the tapered section, and two zones with distinct characteristics (DMLZ and LDZ) were formed near the contact surface with the die. Detailed analyses focusing on the grain shapes of each deformation structure clarified their formation mechanisms and suggested that the formation of the DLMZ influences plastic flow until the deformation reaches a steady state.
3. The evolution of plastic flow during non-steady deformation was quantitatively evaluated by analyses based on elliptical approximations of grains. Changes in the nanohardness maps associated with work hardening corresponded to the evolution of plastic flow. The results of these analyses suggested that an increase in the effective extrusion ratio up to the maximum force, followed by a decrease thereafter, gives rise to the peak of force.

## Data Availability

The original contributions presented in this study are included in the article. Further inquiries can be directed to the corresponding author.
